# Model Development and Validation of Personal Exposure to Volatile Organic Compound Concentrations

**DOI:** 10.1289/ehp.0900561

**Published:** 2009-06-23

**Authors:** Juana Mari Delgado-Saborit, Noel J. Aquilina, Claire Meddings, Stephen Baker, Roy M. Harrison

**Affiliations:** Division of Environmental Health and Risk Management, School of Geography, Earth, and Environmental Sciences, University of Birmingham, Edgbaston, Birmingham, United Kingdom

**Keywords:** benzene, 1,3-butadiene, microenvironment, model, personal exposure, time-weighted model, validation, volatile organic compounds

## Abstract

**Background:**

Direct measurement of exposure to volatile organic compounds (VOCs) via personal monitoring is the most accurate exposure assessment method available. However, its wide-scale application to evaluating exposures at the population level is prohibitive in terms of both cost and time. Consequently, indirect measurements via a combination of microenvironment concentrations and personal activity diaries represent a potentially useful alternative.

**Objective:**

The aim of this study was to optimize a model of personal exposures (PEs) based on microenvironment concentrations and time/activity diaries and to compare modeled with measured exposures in an independent data set.

**Materials:**

VOC PEs and a range of microenvironment concentrations were collected with active samplers and sorbent tubes. Data were supplemented with information collected through questionnaires. Seven models were tested to predict PE to VOCs in 75% (*n* = 370) of the measured PE data set, whereas the other 25% (*n* = 120) was used for validation purposes.

**Results:**

The best model able to predict PE with independence of measurements was based upon stratified microenvironment concentrations, lifestyle factors, and individual-level activities. The proposed model accounts for 40–85% of the variance for individual VOCs and was validated for almost all VOCs, showing normalized mean bias and mean fractional bias below 25% and predicting 60% of the values within a factor of 2.

**Conclusions:**

The models proposed identify the most important non-weather-related variables for VOC exposures; highlight the effect of personal activities, use of solvents, and exposure to environmental tobacco smoke on PE levels; and may assist in the development of specific models for other locations.

Exposure to volatile organic compounds (VOCs) is ubiquitous and can result in a wide range of acute and chronic health effects, such as sensory irritation, nervous system impairment, asthma, and cancer (Caprino and Tonga 1998). Epidemiologic studies play an important role in investigating the health effects of air pollution and are one of the principal bases for setting regulations to protect the public against adverse health effects. In epidemiologic studies, personal exposures (PEs) are estimated from *a*) centrally located monitors, *b*) from the combination of fixed-point monitors in individual microenvironments and activity data defining the times spent in each of the microenvironments, or *c*) from PE monitoring (Leung and Harrison 1998; Payne-Sturges et al. 2004).

Direct measurement of human exposure to VOCs via personal monitoring is the most accurate exposure assessment method currently available. However, its wide-scale application to evaluating exposures at the population level is prohibitive both in terms of cost and time, and sometimes even impractical for certain subpopulations (Liu et al. 2007). Using centrally located monitors has a tendency to underestimate exposures (Serrano-Trespalacios et al. 2004). Consequently, indirect measurements via a combination of microenvironment concentrations and personal activity diaries represent a potentially useful alternative.

Earlier studies showed that modeled PEs can provide a good prediction of overall measured PE (Dodson et al. 2007). Therefore, microenvironment modeling offers an effective mean of estimating population exposures to these pollutants without the considerable logistical difficulties of personal sampling.

None of the previous proposed time-weighted models required PE measures, that is, subjects carrying personal samplers; however, all these models still required measuring concentrations in subject-related environments. Therefore, collecting microenvironment samples was still required to inform these models. A model that does not require direct PE or microenvironment sampling, which uses only lifestyle information collected in questionnaires and a proposed range of microenvironment concentrations structured in different strata such as traffic burden, environmental tobacco smoke (ETS) exposure, and season, has not yet been proposed in the literature. Moreover, increasing the understanding of the variety of factors that influence microenvironment concentrations and hence PEs to air pollutants may lead to improved exposure assessment for use in large-scale epidemiologic studies in which individual measurements are not feasible (Nethery et al. 2008). Consequently, a model that uses large numbers of individual and microenvironment samples under different conditions is able to extrapolate exposures to the general population and hence propose microenvironment concentrations that reflect the range of variability in pollutant concentrations over space and time for key microenvironments would be useful to allow a probabilistic approach in the use of exposure models (Harrison et al. 2002) that does not require subjects’ personal or micro-environment measurements.

So far, it is known that the determination of an individual’s exposure to air pollution depends on the locations where individuals spend time; the individual activity patterns, which are reflected in the time spent in different microenvironments; the type of activities in which individuals are involved (Dodson et al. 2007; Harrison et al. 2002); sociodemographics that define time/activity patterns (Edwards et al. 2006) and environmental factors such as seasonality and community/area effect (Sexton et al. 2004). Some studies have quantified the level of contribution of each microenvironment or activity to the total PE in the general non-occupationally exposed population and have identified sources affecting PEs (Edwards et al. 2001; Kim et al. 2002; Loh et al. 2006).

Although previous studies have shed light upon the distribution of concentrations for PE and much work has been conducted toward modeling population exposures to air pollutants using information collected in time/ activity diaries and microenvironment concentrations, very little has been done toward validating such models at the level of the individual. Because PE models can be useful to assess potential public health impacts from VOCs and to assist in the development of environmental policies to reduce human exposures to and risks from VOCs, it is important to know how well exposure models estimate PE. Therefore, the validation of models with independent data sets is useful to check whether the proposed models serve as surrogates for PE concentrations and to know the extent of the exposure estimation error, which should be accounted for in epidemiologic studies and risk assessments (Liu et al. 2007).

The MATCH project sought to help advance our understanding of the causes and magnitude of exposures to VOC and to establish whether collecting lifestyle information is sufficient to model PEs reliably compared with exposures evaluated independently by personal samplers. In this article, we present the results of model development and validation for 15 VOCs. We propose several models and estimate the confidence with which PEs can be reconstructed using measured microenvironment concentrations. A range of activities affecting VOC concentrations and PEs are identified from information contained on the models and the effect of these activities on PEs is quantified for a U.K.-based nonsmoking non-occupationally exposed population.

## Materials and Methods

### Volunteer recruitment

The MATCH project recruited 100 healthy nonsmoking adult subjects between 2005 and 2007 for non-occupational (i.e., not exposed at workplace) PE in three different areas of the United Kingdom: London, West Midlands, and rural south Wales, where higher, intermediate, and lower exposures were expected. Subjects were chosen to participate based upon four key determinants: the location of their residence (38% urban, 42% suburban, and 20% rural), whether the residence incorporated an integral garage (IG; IG = 16%; non-IG = 84%), the proximity of the residence to a major road [coded as first line (FL); FL = 44%, non-FL = 56%], and whether they were exposed to ETS (ETS = 32%; non-ETS = 68%).

Subjects recruited gave their consent to join the study. Approval was secured for this study from the South Birmingham Research Ethics Committee (REC ref. no. 04/ Q2707/152).

### Sampling methods

PE samples were collected jointly with subject-related micro-environment samples (i.e., home and work-place) and samples in other microenvironments that members of the public visit during their daily activities. All samples were collected actively with pumps connected to sorbent tubes. Details on the list of compounds, sorbent materials, sampling duration, sampling times, and flow rates are available [see Supplemental Material (available online at doi:10.1289/ ehp.0900561.S1 via http://dx.doi.org/)].

### Data collection

In addition to the atmospheric sampling, information related to the subjects was gathered. The subjects filled questionnaires collecting information about subject demographics, lifestyle, home description, products stored within the house, activities performed, places visited, ventilation, and ETS presence, as described in detail elsewhere (Harrison et al. 2009). Further details on the subject demographic characteristics and activities performed during the sampling campaign are available [see Supplemental Material, Table 1 (doi:10.1289/ehp.0900561.S1)].

### Analytical methods

1,3-Butadiene was sampled and analyzed separately from the other VOCs because of its high volatility. Briefly, the VOC and 1,3-butadiene methods comprised the thermal desorption of the compounds and subsequent analysis by gas chromatography and mass spectrometry detection. Further details of the analytical methodology as well as the quality assurance and quality control procedures are available [see Supplemental Material (available online at doi:10.1289/ehp.0900561.S1)].

### Model development

All the data collected in this study, that is, PE (*n* = 500) and microenvironment concentrations (*n* = 510), were used to develop and validate models for predicting PEs. The PE data were split into two different and independent data sets. The first set contains 75% of the data and was used for training the model. The other 25% of the data was saved in order to validate the model developed with the training data set. Samples included in each data set (training and validation) were chosen from the 500 PE data set in order to have a similar distribution of high and low cases in each data set [see Supplemental Material, Table 2 and Figure 1 (doi:10.1289/ ehp.0900561.S1)]. Because home and work-place microenvironment concentrations were not measured for all subjects, the training data set contained 58 cases in model 1, 40 cases in models 2 and 3, and 370 cases in models 4–7, representing in all cases 75% of the available data. The validation data set contained 19 cases in model 1, 10 cases in models 2 and 3, and 120 cases for models 4–7.

Some extreme cases became immediately clear in the training data set. They were characterized by extremely high real concentrations (e.g., PE_benzene_ = 30.2 μg/m3) that could not be modeled using the information provided by the subjects. These cases were classified as outliers considering that the data was three times higher than the top of the interquartile range. The number of outlier cases represented 3.2%, 1.8%, and 1.6% for hexane, toluene, and the rest of the compounds, respectively. These results have been excluded in all the models developed.

Seven different models have been developed using 75% of the data set (i.e., training data set) and tested in 25% of the data set (i.e., validation data set) in order to develop and find the model that best predicts PE to selected VOC. The models developed fall into two distinct categories: empirical (i.e., statistical) models and mechanistic models. Models 1, 2, 3, and 7 are empirical; models 4 and 5 are mechanistic based on time/microenvironment/activity data. Model 6 is a hybrid, which contains a simple mechanistic core (model 5) plus additional empirical terms. Each model proposed and tested is described below.

#### Models 1, 2, and 3

These models assess associations between PE and microenvironment time-weighted concentrations measured in subject’s homes (model 1; Equation 1), workplaces (model 2; Equation 2), and the combination of subject’s home and workplace (model 3; Equation 3) directly related to each subject. The equations used in these models are as follows:


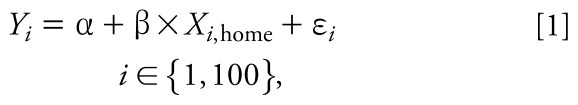



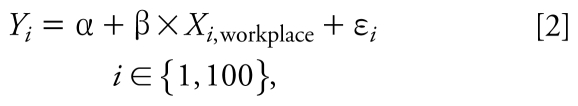



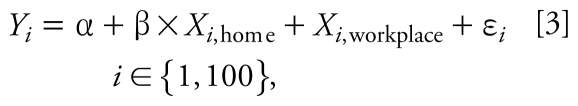


where *Y_i_* is the average of five 24-hr time-weighted measured PE (microgram per cubic meter) samples for subject *i*, α is the intercept of the model, β and γ are the slope of the model, *X_i_*_,home_ is the 24-hr time-weighted participant-specific home concentration (microgram per cubic meter) for subject *i*, and *X_i_*_,workplace_ is the 8-hr time-weighted participant-specific workplace concentration (microgram per cubic meter) for subject *i*. The terms ɛ*_i_* are random error. These models were applied to the available data, which represent 58 cases and 30 cases of the PE data for model 1 and for models 2 and 3, respectively, as a consequence of not having sampled home and workplace microenvironments for all the subjects.

#### Model 4

This model predicts the PE by integrating the time fraction spent in each microenvironment times the concentration of each microenvironment visited:


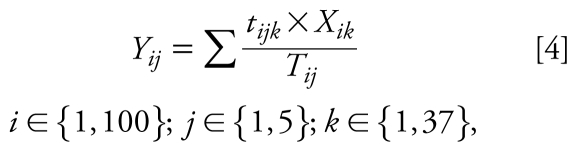


where *Y_ij_* is personal 24-hr predicted exposure (microgram per cubic meter) for subject *i* on day *j*, *t_ijk_* is the time (minutes) spent in microenvironment *k* by subject *i* on day *j*, *X_ik_* is the concentration (microgram per cubic meter) representative of microenvironment *k* for subject *i*, and *T_ij_* is the total time (minutes) spent in all different microenvironments for subject *i* on day *j*, which in turn is the same as the sampling time for subject *i* on day *j*. The basic integrating time fraction used in the questionnaires is 15 min.

The microenvironment concentrations used in model 4 for homes and workplaces were the data collected directly in each subject’s home and workplace. For those volunteers, where no data for home (23%) or work (60%) were available and for all the rest of the microenvironments (streets, transport, and other indoor microenvironments), an average concentration representative of each microenvironment was used. In this study, samples from 37 different microenvironments were collected. The concentration value for each microenvironment applied in model 4 was obtained averaging the concentrations measured in each specific microenvironment. The list of microenvironments measured and each representative concentration are available [see Supplemental Material, Table 3 (doi:10.1289/ ehp.0900561.S1)].

#### Model 5

This model predicts the PE by integrating the time fraction spent in each microenvironment times the concentration of each microenvironment visited as reflected in Equation 5. The difference with model 4 is that, in this case, *a*) the microenvironment concentrations for homes and workplaces were not the data collected directly in each volunteer’s home and workplace, but rather a pooled value representative of the microenvironment (*X_k_*), and *b*) stratified microenvironment concentrations [see Supplemental Material, Tables 4 and 5 (doi:10.1289/ ehp.0900561.S1)] are used instead of a unique value per microenvironment type:


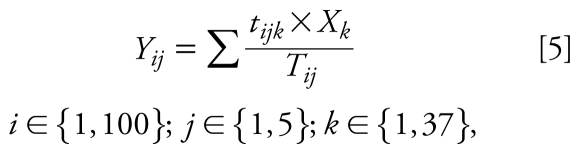


where *Y_ij_*, *t_ijk_*, and *T_ij_* were defined as in Equation 4, and *X_k_* is the concentration (microgram per cubic meter) representative of microenvironment *k*.

For this purpose, a detailed list of stratified microenvironment concentrations (*X_k_*) has been developed for all the microenvironments: homes, workplaces, streets, transport, and other indoor microenvironments. The strata considered were season (i.e., summer/winter), location (i.e., urban/suburban/rural/nonurban), traffic exposure (FL/non-FL), integral garage (IG/no IG), and ETS (ETS/no ETS) as appropriate. Each microenvironment contains different levels of strata and, consequently, different concentrations corresponding to each combination of strata. The concentration value for each combination of strata was the arithmetic mean of the concentration measured in all the samples in each specific combination of strata [see Supplemental Material, Tables 4 and 5 (doi:10.1289/ehp.0900561.S1)].

#### Model 6

This model predicts the PE by integrating the time fraction spent in each microenvironment times the concentration of each microenvironment visited, and also accounts for external factors that might affect exposure, as add-on variables:


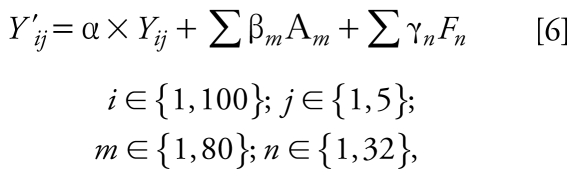


where *Y′_ij_* is the observed 24-hr PE (microgram per cubic meter) for subject *i* on day *j*, *Y_ij_* is the predicted 24-hr PE for subject *i* on day *j* as calculated in model 5 (microgram per cubic meter), α is the coefficient associated with PE *Y_ij_*, *A_m_* are different explanatory variables describing activities performed on day *j* by subject *i* or characteristics associated with volunteer *i*, β*_m_* is the coefficient associated with the explanatory variable *A_m_*, *F_n_* represents the time spent in doing different activities, and γ*_n_* is the coefficient associated to the factor *F_n_*. The explanatory variables, *A_m_*, related to activities (e.g., burning incense) and characteristics (e.g., existence of integral garage) were dichotomous variables, whereas variables representing time (e.g., time exposed to ETS), *F_n_*, were measured in minutes. All the variables (i.e., 80 dichotomous and 32 time-dependent) were extracted from the questionnaires. Seasonality was included in the model as a variable (i.e., summer sampling) and as one of the strata classifying the concentrations for non-subject-related microenvironments [see Supplemental Material, Tables 4 and 5 (doi:10.1289/ehp.0900561. S1)]. The model was developed with SPSS (Chicago, IL, USA), version 15.0, using stepwise linear regression, and further scrutiny of the selected variables for scientific meaning was applied.

#### Model 7

This model predicts PE by focusing explicitly on the factors initially set as key determinants: traffic effect (FL), integral garage (IG), ETS exposure, and living in an urban area, suburban, or rural area:


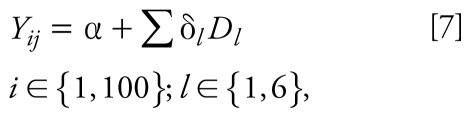


where *Y_ij_* is the observed 24-hr PE for subject *i* on day *j* (microgram per cubic meter), *a* is the constant, *D_l_* is the key determinant as outlined above, and δ*_l_* is the coefficient associated with key determinant *D_l_*. The model was developed with SPSS 15.0 using the Stepwise option from the Linear Regression menu.

To assess the improvements made in the method development and to assess the bias in the prediction, linear regression of PE measured versus predicted in the training set was used and the coefficient of determination (*R*^2^), adjusted coefficient of determination (*R*^2^_adj_), standard error of estimate, and analysis of variance of the regression were checked.

### Model validation

To demonstrate the validity of the parameters estimated in the training data set in other situations within the same experimental conditions, all the proposed models were later validated with the independent data set. Therefore, the developed prediction models (i.e., same regression coefficients obtained in the training data set) were used to predict concentrations in the validation data set. Concentrations measured from the validation data set (25%) and predicted with each model were compared, and the coefficient of determination (*R*^2^), the normalized mean bias (NMB; Equation 8), the mean fractional bias (MFB; Equation 9), and the percentage of predicted values within a factor of 2 (FA2) and a factor of 3 (FA3) were calculated (Briggs et al. 2000).


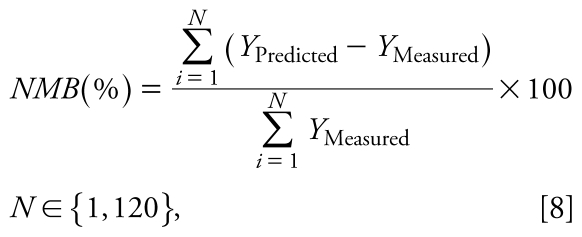



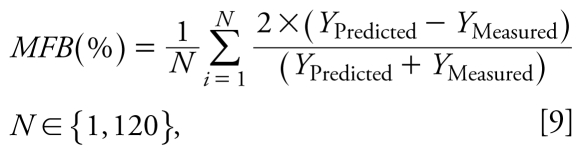


where NMB(%) is the percentage of normalized mean bias, MFB(%) is the mean fractional bias, *Y*_Predicted_ is the concentration predicted with the selected model, *Y*_Measured_ is the concentration measured, and *N* is the total number of cases in the validation data set.

For the mechanistic models (models 4 and 5), the validation approach cannot be extrapolated because no experimental parameter is derived from the exposure data. However, as a homogeneous criterion, the validation procedure has also been performed with these models.

## Results

We used the PE [see Supplemental Material, Table 2 (doi:10.1289/ehp.0900561.S1)] and microenvironment concentrations [see Supplemental Material, Tables 3–5 (doi:10.1289/ehp.0900561.S1)] measured in this study to develop models for prediction of VOC PEs. PE concentrations pertaining to the training data set (75% of the data), subject-related microenvironment concentrations such as home and workplace concentrations, and subject lifestyle information gathered through questionnaires have been integrated into several models in order to predict VOC concentrations in the general population. Table 1 presents the results of the seven models proposed.

The model that correlates PE with home microenvironment (model 1) generates average coefficients of determination (*R*^2^_adj_) of 0.4, whereas for some compounds such as benzene and toluene, the *R*^2^ can be as high as 0.7. On the other hand, the model that correlates PE with the workplace microenvironment (model 2) explains less of the variance in PE, presenting *R*^2^_adj_ in the range of −0.01 to 0.143 for most of the compounds. Model 3, which combines home and work-place measurements, in this study explains less variability than does model 1, which considers only home concentrations, except for 1,3-butadiene, *p*-isopropyltoluene, styrene, and ethylbenzene. However, for none of these compounds is the addition of the workplace concentration significant at the 0.10 level.

The time-weighted model that uses specific subject-related information when available or pooled data for the rest of the cases (model 4) gave an average *R*^2^ of 0.5. On the other hand, model 5, which uses the same approach of time-weighted concentrations, but uses generic stratified microenvironment concentrations instead of direct measurement in individual subjects’ microenvironments, performed worse than model 4. The *R*^2^_adj_ obtained in model 5 ranged from 0.01 to 0.31.

Model 6, which does not require direct measurements in individual subject microenvironments, predicts the personal concentration considering the PE concentration calculated as in model 5 and incorporating add-on variables extracted from the questionnaires that explain additional factors. From all the 112 add-on variables, *A_m_* and *F_n_*, only a range of 3 to 12 variables were selected for each proposed model. Table 2 shows the list of the proposed variables for a subsample of VOC compounds. A correlation analysis between the variables entered into the model was performed to assess collinearity problems in the proposed variables. None of the variables included in the model had Pearson correlations > 0.9 or variance inflation factor > 10.

Model 7, which predicts PE using the key determinants defined in the recruitment strategy [see Supplemental Material, Table 9 (doi:10.1289/ehp.0900561.S1)], was the worst-performing model, with *R*^2^ ranging from 0.01 to 0.27.

Some of the models presented in this study were driven by skewed distributions. Hence, we performed a sensitivity analysis, which consisted of comparing predicted versus measured data in the logged database [see Supplemental Material, Table 6 (doi:10.1289/ehp.0900561. S1)]. This analysis showed that in all cases *R*^2^ values were lower in the logged than in the unlogged version. On the other hand, analyzing the *R*^2^ values in the logged database, models 4 and 6 performed similarly for compounds such as benzene, ethylbenzene, and the xylenes, whereas predicted values with model 4 are slightly better for ETS compounds (pyri-dine and 3-ethenylpyridine), trimethylbenzenes, and toluene. However, model 6 was preferred as the model that better predicted PE within the training data set, because it uses a range of tabulated stratified microenvironment concentrations instead of direct measurements in the subject’s individual microenvironments.

To test the uncertainty associated to the model development and the influence of selecting the 75% of the data for the training data set to provide a similar exposure to that of the validation data set, we performed a second sensitivity analysis. This consisted in selecting three random combinations of 75% of the data for the training data set and producing new models. The results of the performance parameters (e.g., *R*^2^) and associated uncertainty [see supplemental material, Table 7 (doi:10.1289/ ehp.0900561.S1)] of the new models show that the performance of the models proposed in the original data set (Table 1) are comparable to other random combinations.

All the models were tested with data contained in the validation data set. In this case, the coefficients of determination (Table 1) are better for models 1–3, worse for models 4 and 5, and similar for models 6 and 7 than those obtained in the training data set. The normalized mean bias (NMB) shows that most of the compounds were overpredicted by around 10% in model 1 and 4–77% in model 2, over- and underpredicted in model 3 (−33% to 37%), and underpredicted by models 4–6, ranging from 15% to 30%. Model 7 over- and underpredicted concentrations showing the higher range of NMB (i.e., 94–768%). The percentage of predicted values within a factor of 2 is around 60–80%, whereas this percentage increases to 80–100% if a factor of 3 is considered for all models except model 7.

## Discussion

The PE and microenvironment concentrations measured in this study are substantially lower than those found in similar studies, conducted in different locations in the United States and Europe and at earlier times (Edwards et al. 2001; Kim et al. 2002; Loh et al. 2006).

This study presents seven different approaches used in the development of models that predict VOC concentrations for non-occupational PEs. Distinct category models have been tested, and each category has its own applicability and limitations. First, models 1–3 use the average of 5-day time-weighted PE concentration, whereas models 4–7 use daily 24-hr time-weighted PE. This difference in time integration scale will smooth the effect of short time activities in PE for models 1–3, so these models will be less affected by day-to-day variations. Second, empirical models (models 1, 2, 3, and 7) can be used for predictions as long as the conditions predicted are similar to those of the original data collection. In principle, empirical models cannot be transferred to new and different conditions, because they contain no information that is not contained in the original data. On the other hand, mechanistic models (model 4 and 5) are based on an assumed and simplified logical construct of the exposure conditions, and therefore can be applied for any conditions as long as the logical construct remains. The hybrid model (model 6) contains features of both mechanistic and empirical models, and therefore, expansion of the model to new conditions (e.g., new country, area, or time) needs specific consideration and possibly some readjustment. Even with all these considerations, valuable information can be drawn out from each of the proposed models.

The information extracted from the performance of models 1–3 gives valuable information in terms of evaluating the influence on PE by the two microenvironments where people spent most of their time. The model that correlates PE with home microenvironment concentrations (model 1) explains most of the variance of the PE. Home concentrations explain 60–75% of the PE variance for compounds such as benzene, toluene, and 3-ethenylpyridine and 40–55% for compounds such as the xylenes, trimethylbenzenes, and pyridine, whereas for compounds such as hexane, styrene, or 1,3-butadiene model 1 explains 5–15% of the variance in PE. On the other hand, workplace VOC concentrations (model 2) are not as good at predicting PE as are home concentrations. This is mainly due to the presence of high concentrations in the PE subset not related to workplace concentration as a consequence of strong VOC sources such as home indoor or personal activity sources. The observation that the home is a stronger predictor is also emphasized by the fact that the model that includes home jointly with workplace concentrations (model 3) does not improve the *R*^2^ coefficient with respect to model 1, and the inclusion of the workplace variable is not significant at the 0.01 level for most of the compounds. This is contrary to what was expected, because PE predictions should have improved when increasing the number of microenvironment concentrations included in the model. As outlined before, this might have been a consequence of a set of PE activities not reflected in workplace concentrations. Therefore, these results suggest that the microenvironment that affects PE most is the home microenvironment, where subjects spent an average of 62% of their time. PE was hence dominated by the contribution from the residential environment for all compounds, as previously reported by other authors (Adgate et al. 2004; Kim et al. 2002).

Two model approaches were based on predicting the PE integrating the time fraction spent in different microenvironments times the microenvironment concentration. The time-weighted model that used microenvironment concentrations measured directly in the homes and workplaces of the subjects (model 4) explained an average variability of 50% ranging from 12% to 80%. Similar results for benzene were reported by Adgate et al. (2004) and for other compounds by Kim et al. (2002). Nevertheless, direct measurements in the subjects’ own locations are required to feed this model. On the other hand, model 5 follows the same approach but uses independent generic stratified microenvironment concentrations for all the microenvironments that the subjects visit. Therefore, no direct measurements are required to predict PE. The performance of this model is lower compared with the other tested models. This was expected as the generic stratified microenvironment concentrations used in model 5 does not contain specific information about each subject’s microenvironment as was the case in model 4. This is a consequence of the difficulties in adequately stratifying home microenvironments with the modest number of samples collected, combined with the home-to-home variation in home concentrations. Even when the total sample size for home microenvironment is large (number of home samples = 77), the large number of different strata, such as integral garage, ETS exposure, first-line (FL) properties, or location within a city, considerably reduces the sample size per stratum. In addition, the range of activities in which the subjects are engaged in their normal life was reflected in the specific home and workplace levels and therefore was well accounted for in models 1 and 4. This, however, does not occur in the same way in the stratified VOC levels representative of each different stratum of exposure proposed in model 5. Examination of the data [see Supplemental Material, Figure 2e (doi:10.1289/ehp.0900561.S1)] shows that model 5 predicts the concentrations well except in some cases where concentrations are in most instances underpredicted. Further the study showed that these cases are linked to activities such as exposure to ETS, do-it-yourself projects, photocopying, use of solvents, and so forth. Therefore, model 5 predicts the concentration well in most instances, but does not perform well when the subjects engage in an activity that implies a substantial increase in VOC exposure.

To solve this difficulty, model 6 uses the concentrations calculated in model 5, but includes a range of add-on variables that represent activities or home characteristics that could not be reflected in the stratified data and that lead to an increase of the VOC levels. This new model approach better reflects the VOC concentrations, explaining higher levels of variance: 80% for ethylbenzene, xylenes, styrene, and trimethylbenzenes and 45–50% for compounds such as benzene, toluene, and 1,3-butadiene. The amount of variance left unexplained by model 6 must be because the sources of such compounds were not well captured in the model by the proposed microenvironment concentrations or add-on variables.

The model that predicted PEs from key determinants (model 7) is the one that explains the least variability of all the seven tested models, implying that for predicting PEs it is advisable to include microenvironment concentration data into the model. This model, although not suited for prediction purposes, gives valuable information in identifying determinants of VOC exposures [see Supplemental Material, Table 9 (doi:10.1289/ ehp.0900561.S1)].

If indirect exposure estimates are to be routinely employed, then it is important that they are evaluated by comparison with an independent dataset of direct measurements. Such comparison has been conducted in the present study, with the authors concluding that there is good agreement between measured and predicted concentrations for most of the models proposed. The models that perform best in both the training and validation data sets are the empirical model 1 and the hybrid model 6, with model 7 the worst-performing model in both data sets.

The hybrid model 6, which includes time-weighted concentrations, independent stratified data, and add-on variables collected from questionnaires, is considered the best-performing model in order to predict PEs while minimizing the cost of direct measurements in the subjects’ homes or workplaces. It is, however, important to recognize that for benzene and toluene the best model is model 1, where PE is predicted directly from home exposure. It appears that for these compounds, the inclusion in the model of other microenvironments visited (models 3–5) or other activities performed during the day (model 6) does not improve the prediction of PEs, but increases the uncertainty not accounted for by the model (e.g., 74%, 44%, and 47% of variability explained for benzene by models 1, 4, and 6, respectively). Most of the variation for these components arises from the home concentration. On the other hand, the other microenvironments or activities that also contribute to the PE could not be captured by the other proposed models (models 4–6). This suggests that more detailed information is needed to fully understand other sources contributing to benzene and toluene concentration in addition to the home microenvironment, and that the estimates of home microenvironment concentrations proposed in the generic stratified data (models 5 and 6) do not fully reflect the behavior of these compounds and hence need further study.

The influence that several activities have on the VOC levels can be assessed from the information contained in model 6 [Table 2; see also Supplemental Material Table 8 (doi:10.1289/ ehp.0900561.S1)]. ETS exposure is important for 3-ethenylpyridine, pyridine, benzene, and 1,3-butadiene. Traffic is a good predictor for compounds such as benzene, toluene, and 1,3-butadiene because all these models contain traffic-related variables. The use of paints is an activity affecting the levels of ethylbenzene, the xylenes, and the trimethylbenzenes. Similarly, storing paints in an integral garage increases the levels of *n*-hexane, benzene, toluene, and ethylbenzene. In line with activities related to the integral garage, parking the car in the garage raises the levels of benzene, toluene, ethylbenzene, the xylenes, and the trimethyl-benzenes. The use of fuels other than natural gas for heating increases the concentrations of *n*-hexane, benzene, ethylbenzene, and *p*-xylene. The variables chosen are supported by the literature, as reported in Table 2 [see also Supplemental Material, Table 8 (doi:10.1289/ ehp.0900561.S1)].

For benzene, the proposed model takes into account the existence of an integral garage where paints and the car are kept, traffic variables such as living in an urban area, use of trains, and time spent commuting by car, ETS exposure, variables related to heating, and other variables linked to activities such as working in a hospital. Storage of paints in the garage has been identified as the strongest predictor, followed by time exposed to ETS and parking of a car in the garage. Higher benzene levels have been previously related to integral garages (Batterman et al. 2006), ETS (Heavner et al. 1995), and traffic (Edwards et al. 2001). As regards 1,3-butadiene, the variables suggested were use of solvents, ETS-related variables, visiting a gasoline station, time since carpet was placed in the living room, time spent commuting, location of the door communicating with the integral garage in the kitchen, use of additional heating other than natural gas and electricity, time spent using sprays for decoration (e.g., artificial snow spray) and wrapping presents, and time since mold was removed from the house. The literature reports transport, prescribed burning, residential and commercial space heating, fuel and gasoline distribution, burning of other materials (e.g., cigarettes), and exposure to ETS as 1,3-butadiene sources (Curren et al. 2006). The information extracted from the models suggests that policies leading to the reduction in the VOC content of products used in the home and in ETS exposures would help reduce PEs to VOC compounds.

Several subjects with particularly high PEs have been identified in this study. The high exposures were attributed to activities within the home and exposures to ETS, which play a major role in determining exposure. Therefore, efforts to reduce PE in the high-exposure group would be best focused upon regulating highly emitting household products and exposure to tobacco smoke. On the other hand, most of the subjects had lower PE deriving largely from indoor concentrations, which in turn are related to outdoor sources via infiltration and air exchange (Turpin et al. 2007). For the general population, abatement measures relating to outdoor sources will have a large relative impact. Because this group is far more numerous, the population-wide health benefits, especially for nonthreshold toxics, deriving from abatement of outdoor sources may be appreciable. Also, reduction in the VOC content of products used in the home and in ETS exposures would have a major benefit in reducing exposure of both groups.

The results of model development obtained in this study are comparable with previously developed models. Heavner et al. (1995) predicted PE to benzene based on dichotomous variables related to ETS and home characteristics as well as continuous variables related to ETS, explaining 28% of the variance. The authors acknowledged that the total sum of all other unidentified benzene sources in the model could have exerted an overall greater effect on benzene concentrations than the variables applied in that model, which is consistent with our findings. Leung and Harrison (1998) estimated 12-hr integrated exposures by combining activity data with measurements performed in subjects’ homes and offices as well as with independent measurements of VOCs in several microenvironments, which is an approach similar to our proposed model 4. They reported better benzene and toluene results (i.e., benzene *R*^2^ = 0.83, toluene *R*^2^ = 0.92), but worse *m*,*p*-xylene results (i.e., *R*^2^ = 0.56). Payne-Sturges et al. (2004) compared the values predicted with the U.S. Environmental Protection Agency model ASPEN (Assessment System for Population Exposure Nationwide) with 3-day average PE measured with passive samplers. For most of the VOCs, the median ratios were comparable within a factor of 2, showing good agreement between ambient measurements and model predictions across the community measured. Dodson et al. (2007) used a series of time-weighted PE models to predict measured PE to several VOCs. The relative percent difference between measured and modeled with the fully saturated time-weighted exposure model, which is a similar approach to our proposed model 4, ranged between 5% and 15%. Perez Ballesta et al. (2008) modeled VOC from time/microenvironment/activity diary information (i.e., model 4 approach) and reported an *R*^2^ value of 0.47 for benzene, which is similar to the value reported in this study, but the *R*^2^ values for toluene (*R*^2^ = 0.32), ethylbenzene (*R*^2^ = 0.27), and *m*,*p*-xylene (*R*^2^ = 0.56) exposure concentrations were lower than those reported in this study. Edwards et al. (2001) modeled VOC concentrations using the time/ microenvironment/activity approach generally underestimating the results between 6% and 15%. Differences between the results of our models and former reported models might also be a consequence of a different range of sources, microenvironments, and temporal and spatial representativeness of the exposed population.

Despite the fact that the previous studies have reported valuable information in terms of understanding how to predict VOC PE from measurements directly made in subject-related microenvironments and time/microenvironment/activity data, none has used independent microenvironment concentrations (i.e., not measured at the subject’s homes or workplaces) or has verified their model results with an independent data set.

From the difficulties observed and lessons learned in the present study, we can make several recommendations for future model development: using a larger set of PE data; using a larger data set of microenvironment concentrations in order to perform an accurate and detailed stratification of the microenvironments entered into the model; having larger variability in PE and in important predictors; gathering more detailed information recorded in the questionnaires about activities performed and microenvironments visited by the subjects; and including detailed meteorologic information.

## Conclusions

Several models that have been developed for predicting PEs and for validating the models with an independent data set suggest that individual-level activity and microenvironmental data are needed for modeling PE when direct subject-related microenvironment sampling is excluded (e.g., no direct sampling in subjects’ home or workplaces). In contrast, the PE model using only housing characteristics (e.g., having an integral garage) was not very effective and the worst performing of the models tested. On the other hand, benzene and toluene PE were better predicted with models that included only direct home microenvironment measurements. This suggests that further study is required to understand the variability of benzene and toluene concentrations at home in order to identify concentrations representative of home microenvironments that can be used in subject-independent prediction models.

The data extracted from the models are indicative of a number of sources making important contributions to the concentrations of VOCs to PEs. Road traffic, the use of solvents, and ETS exposure make important contributions. It has been observed that where ETS is present in any environment it causes increases in a wide range of VOCs. Specific activities of the subjects and those around them can lead to elevated exposures, and many of these have been identified. However, even when included in models of PE, these do not account for the full exposure to some compounds, so some exposure sources appear not to have been recognized.

Finally, although the proposed models identify the most important non-weather-related variables for VOCs, the use of such models in different geographical regions, countries, times, climates, and locations with markedly different sources of pollutants will require caution. Therefore, the use of empirical or hybrid models may need some adjustment to the specific conditions for new exposure scenarios, whereas the mechanistic model approach would be applicable to any situation that considers time-weighted exposures. Yet, the models presented in this study will serve as a base and guide to design studies in order to develop specific models for different locations.

## Figures and Tables

**Table 1:** *R*^2^, *R*^2^_adj_, SE (μg/m3), NMB, MFB, and FA2 and FA3 in training (TR) and validation (VAL) data sets.

Model/ data set	Hexane	Benzene	Toluene	Ethyl-benzene	*p*-Xylene	*m*-Xylene	Pyridine	*o*-Xylene	1,3,5-Tri- methyl- benzene	Styrene	*p*-Isopropyl- toluene	1,2,4-Tri- methyl- benzene	3-Ethenyl-pyridine	Naphth-alene	1,3-Buta- diene
Model 1															
TR															
*R*^2^	0.15^a^	0.74^a^	0.66^a^	0.23^a^	0.49^a^	0.48^a^	0.39^a^	0.48^a^	0.49^a^	0.05	0.30^a^	0.54^a^	0.61^a^	0.20^a^	0.11^a^
*R*^2^_adj_	0.13^a^	0.74^a^	0.65^a^	0.21^a^	0.48^a^	0.47^a^	0.38^a^	0.47^a^	0.48^a^	0.03	0.29^a^	0.54^a^	0.61^a^	0.19^a^	0.09^a^
SE	2.65	0.76	8.58	2.28	1.51	3.73	0.20	1.94	1.29	0.33	0.62	3.77	0.31	0.61	36
VAL															
*R*^2^	0.65^a^	0.83^a^	0.89^a^	0.86^a^	0.69^a^	0.59^a^	0.71^a^	0.63^a^	0.94^a^	0.86	0.47^a^	0.95^a^	0.98^a^	0.65^a^	0.02^a^
NMB (%)	−25	0	5	−18	7	2	−4	6	12	−47	17	7	4	8	−45
MFB (%)	7	7	33	34	34	29	23	29	−12	52	25	15	60	29	−47
FA2 (%)	35	100	65	65	75	60	75	70	75	45	80	80	60	75	15
FA3 (%)	65	100	85	85	85	90	90	85	85	85	95	100	70	100	20

Model 2															
TR															
*R*^2^	0.10	0.12	0.17	0.05	0.08	0.06	0.00	0.07	0.01	0.01	0.05	0.01	0.01	0.04	0.05
*R*^2^_adj_	0.07	0.10	0.14	0.02	0.05	0.03	0.03	0.04	−0.01	−0.01	0.03	−0.02	−0.01	0.01	0.02
SE	2.99	1.44	12.9	1.73	1.97	4.6	0.22	2.27	1.08	0.32	0.47	3.87	0.34	0.74	0.21
VAL															
*R*^2^	0.28	0.17	0.18	0.70	0.25	0.69^a^	0.00	0.23	0.48^a^	0.28	0.01	0.32	0.03	0.31	0.00
NMB (%)	−2	9	14	35	56	37	−16	61	70	4	17	77	−34	38	−35
MFB (%)	41	14	30	43	50	43	48	50	62	14	29	69	46	38	38
FA2 (%)	50	88	75	75	63	75	75	63	50	88	75	38	50	75	25
FA3 (%)	63	100	75	88	88	88	75	88	50	100	88	63	75	100	38

Model 3															
TR															
*R*^2^	0.17	0.74^a^	0.65^a^,^b^	0.38^a^	0.39^a^,^b^	0.37^a^	0.21^a^	0.36^a^,^b^	0.44^a^	0.27^a^	0.39^a^	0.49^a^	0.43^a^	0.04	0.56^a^
*R*^2^_adj_	0.12	0.72^a^	0.63^a^,^b^	0.34^a^	0.36^a^,^b^	0.33^a^	0.16^a^	0.32 a,b	0.41^a^	0.23^a^	0.35^a^	0.46^a^	0.39^a^	−0.01	0.52^a^
SE	2.97	0.82	8.70	1.45	1.65	3.90	0.21	1.96	0.86	0.28	0.39	2.91	0.27	0.78	0.15
VAL															
*R*^2^	0.34	0.25	0.67^a^	0.93^a^	0.57^a^	0.91^a^	0.37	0.54^a^	0.81^a^	0.37	0.05	0.83^a^	0.93^a^	0.67^a^	0.85^a^
NMB (%)	−5	−1	4	23	36	25	2	37	21	−1	−2	20	−1	31	−33
MFB (%)	40	4	21	36	38	35	60	36	27	7	12	29	66	38	31
FA2 (%)	50	88	75	75	63	75	63	75	88	88	75	100	63	75	38
FA3 (%)	50	100	88	88	100	88	75	100	100	100	100	100	75	100	38

Model 4															
TR															
*R*^2^	0.51^a^	0.44^a^	0.53^a^	0.56^a^	0.63^a^	0.60^a^	0.24^a^	0.63^a^	0.62^a^	0.79^a^	0.43^a^	0.67^a^	0.30^a^	0.79^a^	0.12^a^
*R*^2^_adj_	0.51^a^	0.44^a^	0.52^a^	0.56^a^	0.63^a^	0.60^a^	0.24^a^	0.63^a^	0.62^a^	0.79^a^	0.43^a^	0.67^a^	0.30^a^	0.79^a^	0.12^a^
SE	1.24	0.89	11.30	0.89	0.91	2.19	0.22	0.98	0.33	0.38	0.35	1.23	0.26	0.38	0.28
VAL															
*R*^2^	0.02	0.16	0.19	0.03	0.00	0.01	0.27	0.00	0.03	0.03	0.02	0.07	0.32	0.00	0.02
NMB (%)	−25	−21	−1	−18	−22	−20	−19	−29	−10	−22	−14	−19	−24	10	−27
MFB (%)	−37	−18	9	−48	−45	−45	−20	−47	−33	24	20	−38	−33	−34	106
FA2 (%)	39	81	73	66	57	58	56	59	58	66	72	56	49	74	33
FA3 (%)	58	97	90	82	75	74	78	78	78	93	88	81	72	90	52

Model 5															
TR															
*R*^2^	0.02	0.16^a^	0.11^a^	0.07^a^	0.13^a^	0.13^a^	0.19^a^	0.15^a^	0.20^a^	0.28^a^	0.05	0.32^a^	0.26^a^	0.13^a^	0.08
*R*^2^_adj_	0.01	0.16^a^	0.11^a^	0.07^a^	0.12^a^	0.12^a^	0.19^a^	0.15^a^	0.19^a^	0.28^a^	0.04	0.31^a^	0.26^a^	0.13^a^	0.07
SE	1.13	0.98	9.81	0.94	1.08	2.60	0.22	1.34	0.33	0.64	0.26	1.28	0.25	0.47	0.41
VAL															
*R*^2^	0.27	0.06	0.12	0.08	0.14	0.11	0	0.1	0.16	0.05	0	0.02	0	0.11	0.06
NMB (%)	−30	−16	−23	−16	−27	−23	−20	−26	−30	−11	−21	2	−29	−19	26
MFB (%)	−27	−16	−19	−27	−42	−39	−50	−44	−30	27	14	14	3	−25	119
FA2 (%)	44	74	51	61	55	52	64	51	58	70	64	54	56	65	31
FA3 (%)	63	88	70	76	73	72	86	74	77	88	83	77	68	83	44

Model 6															
TR															
*R*^2^	0.39^a^	0.47^a^	0.51^a^	0.81^a^	0.82^a^	0.83^a^	0.70^a^	0.83^a^	0.79^a^	0.87^a^	0.48^a^	0.86^a^	0.75^a^	0.42^a^	0.49^a^
*R*^2^_adj_	0.38^a^	0.45^a^	0.50^a^	0.81^a^	0.82^a^	0.83^a^	0.70^a^	0.81^a^	0.78^a^	0.87^a^	0.47^a^	0.81^a^	0.75^a^	0.41^a^	0.48^a^
SE	2.85	0.97	12.31	1.43	1.42	3.39	0.23	1.67	1.24	1.18	0.72	3.31	0.34	0.9	0.39
VAL															
*R*^2^	0.48^a^	0.44^a^	0.53^a^	0.64^a^	0.62^a^	0.52^a^	0.66^a^	0.62^a^	0.71^a^	0.94^a^	0.36^a^	0.83^a^	0.61^a^	0.06	0.08
NMB (%)	−25	−20	−7	−10	−15	−23	−6	14	−22	8	−6	−28	−39	17	−40
MFB (%)	44	−8	14	6	11	4	25	41	−24	16	13	−7	16	23	27
FA2 (%)	43	80	61	73	69	66	63	56	49	57	76	60	42	64	31
FA3 (%)	61	92	83	87	90	87	75	84	72	84	91	83	62	84	49

Model 7															
TR															
*R*^2^	0.13^a^	0.09^a^	0.02^a^	0.01	NVE	0.01	0.22^a^	0.02	0.04^a^	0.01	NVE	0.05^a^	0.27^a^	0.05^a^	0.09^a^
*R*^2^_adj_	0.12^a^	0.08^a^	0.02^a^	0.01	NVE	0.01	0.21^a^	0.01	0.04^a^	0.01	NVE	0.04^a^	0.27^a^	0.04^a^	0.09^a^
SE	3.38	1.25	17.17	3.25	NVE	8.16	0.36	4.46	2.61	3.21	NVE	7.42	0.59	1.16	0.5
VAL															
*R*^2^	0.08	0.18	0.05	0.00	0.03	0.05	0.00	0.03	0.01	0.01	0.01	0.04	0.44	0.05	0.01
NMB (%)	−31	−21	1	−96	140	−94	768	−56	45	144	−52	−70	−7	−22	23
MFB (%)	15	−10	12	−92	80	−153	163	−38	36	92	−93	−60	53	−28	67
FA2 (%)	37	87	57	39	13	1	1	43	33	24	28	55	39	48	45
FA3 (%)	67	94	79	54	30	9	6	70	61	34	37	75	55	81	63

NVE, no variable entered in the stepwise regression. Sample sizes (training/validation data sets): model 1, 58/19; models 2 and 3, 40/10; models 4–7, 370/120.

a Represent significant correlation values at 0.01 level.

b The workplace concentration coefficient γ is significant at the 0.01 level.

**Table 2:** Detailed list of add-on variables (*A_m_* and *F_n_*), unstandardized variable coefficients (β*_m_* and γ*_n_*), standardized variable coefficient (β), *t*-statistic and its significance (Sig) and partial correlations of the variables entered in the development of model 6, as defined in Equation 6.

Model	Variables	Unstandardized coefficients (β*_m_*, γ*_n_*)		Standardized coefficient	*t*-Statistic	Sig	Correlations			Percent cases report add-on	Reference supporting variable
		β*_m_* or γ*_n_*	SE	β			Zero order	Partial	Semipartial		
Benzene (*R*^2^ = 0.469)	(Constant)	0.609	0.196		3.108	0.002					
	Benzene modeled	0.140	0.082	0.096	1.696	0.091	0.316	0.093	0.068	100	

	Dichotomous variables *A_m_*										
	Volunteer has paints stored in the garage	1.905	0.210	0.405	9.082	0.000	0.464	0.447	0.364	7	
	Car kept in the garage	1.049	0.215	0.219	4.889	0.000	0.382	0.260	0.196	7	Batterman et al. 2006
	Urban location	0.380	0.133	0.140	2.854	0.005	−0.094	0.155	0.114	38	Edwards et al. 2005
	Volunteer visited hospital	1.320	0.316	0.170	4.171	0.000	0.183	0.223	0.167	3	
	Commute by electric or diesel train	0.350	0.179	0.083	1.962	0.051	0.023	0.107	0.079	10	Edwards et al. 2005
	Gas main heating	0.455	0.123	0.172	3.714	0.000	0.032	0.200	0.149	3	
	Additional heating sources	2.087	0.668	0.148	3.122	0.002	0.175	0.169	0.125	60	

	Time variables *F_n_*										
	Time exposed to constant or frequent ETS	0.003	0.001	0.279	5.994	0.000	0.285	0.313	0.240	15	Heavner et al. 1995
	Time commuting in a car	0.005	0.001	0.153	3.597	0.000	0.243	0.194	0.144	11	

Ethylbenzene (*R*^2^ = 0.813)	(Constant)	0.981	0.198		4.944	0.000					
	Ethylbenzene modeled	0.156	0.156	0.032	0.995	0.320	0.117	0.055	0.024	100	

	Dichotomous variables *A_m_*										
	Volunteer has paints stored in the garage	1.668	0.309	0.142	5.393	0.000	0.183	0.284	0.128	7	Ilgen et al. 2001
	Car kept in the garage	1.807	0.321	0.152	5.637	0.000	0.159	0.296	0.134	7	Song et al. 2007
	Volunteer visited hospital	2.113	0.463	0.109	4.559	0.000	0.088	0.243	0.108	3	Song et al. 2007
	Carpet has been fumigated	19.158	0.836	0.546	22.929	0.000	0.525	0.783	0.545	1	Yang et al. 2002
	New carpet	0.202	0.200	0.025	1.006	0.315	−0.015	0.055	0.024	1	
	Volunteer works in a factory	3.881	0.836	0.111	4.645	0.000	0.085	0.247	0.110	3	Song et al. 2007
	Additional heating sources	3.113	1.057	0.089	2.945	0.003	0.082	0.160	0.070	1	Song et al. 2007

	Time variables *F_n_*										
	Time painting or in presence of painters	0.074	0.003	0.675	28.345	0.000	0.662	0.841	0.674	3	Ilgen et al. 2001, Song et al. 2007

Styrene (*R*^2^ = 0.868)	(Constant)	0.209	0.171		1.224	0.222					
	Styrene modeled	0.796	0.248	0.064	3.208	0.001	0.035	0.172	0.064	100	

	Dichotomous variables *A_m_*										
	Wood burning in fireplace	2.503	0.489	0.102	5.117	0.000	0.094	0.268	0.102	2	Austin et al. 2001
	Carpet has been fumigated	31.987	0.688	0.926	46.475	0.000	0.922	0.930	0.925	1	Zuraimi et al. 2006

3-Ethenylpyridine (*R*^2^ = 0.750)	(Constant)	0.109	0.022		4.912	0.000					
	3-Ethenylpyridine modeled	−0.337	0.052	−0.318	−6.472	0.000	0.370	−0.333	−0.172	100	

	Dichotomous variables *A_m_*										
	ETS	0.178	0.051	0.116	3.482	0.001	0.468	0.187	0.092	30	Heavner et al. 1995

	Time variables *F_n_*										
	Time in presence of ETS	0.001	0.000	0.227	3.970	0.000	0.761	0.212	0.105	30	Heavner et al. 1995
	Time exposed to constant ETS	0.003	0.000	0.215	5.204	0.000	0.711	0.273	0.138	6	Heavner et al. 1995
	Time exposed to constant or frequent ETS	0.004	0.000	0.647	9.808	0.000	0.777	0.472	0.260	15	Heavner et al. 1995

Naphthalene (*R*^2^ = 0.418)	(Constant)	0.381	0.082		4.659	0.000					

	Dichotomous variables *A_m_*										
	Incense burning	0.994	0.248	0.168	4.006	0.000	0.146	0.214	0.167	2	
	Photocopier, fax, or printer in the house	0.306	0.135	0.097	2.272	0.024	0.205	0.123	0.095	18	Zuraimi et al. 2006
	Gas main heating	0.273	0.102	0.115	2.671	0.008	0.045	0.145	0.111	59	
	Packing or unpacking clothes	1.188	0.906	0.055	1.311	0.191	0.040	0.072	0.055	1	Edwards et al. 2005

	Time variables *F_n_*										
	Inverse time carpet was replaced in kitchen	0.921	0.693	0.058	1.329	0.185	0.068	0.073	0.055	7	
	Inverse time carpet was replaced in living room	0.164	0.333	0.021	0.493	0.622	0.001	0.027	0.021	7	
	Inverse time packing or unpacking clothes	20.078	1.435	0.600	13.991	0.000	0.603	0.608	0.584	1	Edwards et al. 2005

1,3-Butadiene (*R*^2^ = 0.487)	(Constant)	0.115	0.036		3.232	0.001					

	Dichotomous variables *A_m_*										
	Visited a gasoline station	0.178	0.106	0.068	1.678	0.094	0.107	0.092	0.066	4	
	Commute by bus	0.108	0.070	0.062	1.538	0.125	0.094	0.084	0.060	4	Curren et al. 2006
	Solvent use	0.112	0.135	0.034	0.834	0.405	0.117	0.046	0.033	13	
	Garage connected to the kitchen	0.401	0.091	0.176	4.403	0.000	0.145	0.235	0.173	5	
	Additional gas heating appliances	0.666	0.112	0.238	5.969	0.000	0.198	0.312	0.234	13	

	Time variables *F_n_*										
	Time in presence of ETS	0.001	0.000	0.149	1.969	0.050	0.389	0.108	0.077	12	Heavner et al. 1995
	Time exposed to constant or frequent ETS	0.001	0.000	0.236	3.150	0.002	0.375	0.171	0.124	30	Heavner et al. 1995
	Time traveling	0.001	0.000	0.059	1.458	0.146	0.042	0.080	0.057	15	Curren et al. 2006
	Time wrapping Christmas presents and cards	0.015	0.001	0.431	10.685	0.000	0.448	0.506	0.419	1	
	Inverse time since antimold use	2.001	0.338	0.241	5.925	0.000	0.247	0.310	0.232	1	

Concentrations are measured in micrograms per cubic meter (μg/m ), time variables are measured in minutes, and inverse of time variables in minutes .
